# Synthesis, X‐Ray Analysis, Anticancer Activity, Computational, and in Silico Studies of New Thiophene Pyrazole Conjugates

**DOI:** 10.1002/open.70226

**Published:** 2026-05-18

**Authors:** Abdullatif Bin Muhsinah, Nabila A. Kheder, Ismail A. Elhaty, Yahia N. Mabkhot

**Affiliations:** ^1^ Department of Pharmacognosy College of Pharmacy King Khalid University Abha Saudi Arabia; ^2^ Department of Chemistry Faculty of Science Cairo University Giza Egypt; ^3^ Department of Nutrition and Dietetics Faculty of Health Sciences Istanbul Gelisim University Istanbul Turkey; ^4^ Department of Pharmaceutical Chemistry College of Pharmacy King Khalid University Abha Saudi Arabia

**Keywords:** anticancer activity, drug‐likeness, pyrazole, thiophene, x‐ray analysis

## Abstract

A convenient synthesis of two new thiophene‐appended pyrazoles, **8a** and **8b**, from thiophene derivative **3** is reported. The structures of the synthesized compounds were confirmed by infrared, nuclear magnetic resonance, and mass spectroscopy analysis, while compound **8a** was further confirmed by single‐crystal X‐ray diffraction and computational studies. The crystal structure of **8a** revealed a nonplanar conformation stabilized by N—H···O hydrogen bonding and a weak C—H···O/N contacts. Hirshfeld surface analysis of **8a** showed that H···H contacts dominate the packing, accounting for 52.8%. Density functional theory calculations showed a highest occupied molecular orbital–lowest unoccupied molecular orbital gap of 3.999 eV, indicating a moderate electronic stability with charge transfer ability. The in vitro antitumor activity of the synthesized compounds was evaluated against liver (HepG2), breast (MCF‐7), and colorectal (HCT‐116) cancer cell lines, using the sulforhodamine B (SRB) assay. Compound **3** showed the highest activity against MCF‐7 (IC_50_ = 2.2 ± 0.3 μg/mL), while the thiophene pyrazole hybrid **8b** demonstrates greater activity than **8a** against all tested cell lines. In silico evaluation indicated that **8b** showed the most balanced safety and drug likeness profile.

## Graphical abstract

Two new thiophene–pyrazole derivatives, **8a** and **8b**, were synthesized from thiophene **3** precursor and fully characterized. The structure of **8a** was confirmed by single‐crystal X‐ray diffraction, which revealed a nonplanar conformation stabilized by N—H···O hydrogen bonding and weak intermolecular contacts. Hirshfeld surface and density functional theory analyses showed that H···H contacts dominate the crystal packing of **8a** with a moderate highest energy occupied molecular orbital–lowest energy unoccupied molecular orbital gap of 3.999 eV, indicating electronic stability with charge transfer ability. Biological evaluation against HepG2, MCF‐7, and HCT‐116 cancer cell lines showed that compound **3** was the most active against MCF‐7, while **8b** displayed a greater activity than **8a** against all tested cell lines. In silico toxicity and drug‐likeness evaluation indicated that **8b** showed the most balanced profile.

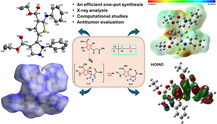

## 1 Introduction

**FIGURE 1:**
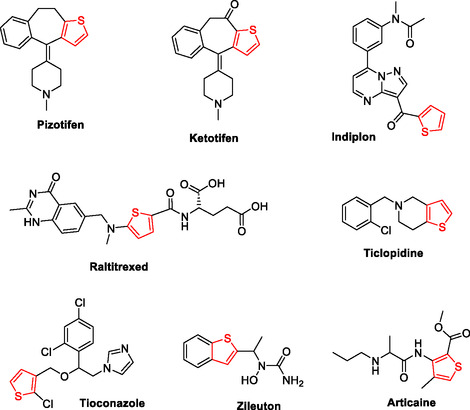
Some marketed medications contain the thiophene moiety.

Several pharmacological activities have been identified for thiophene‐containing compounds, including antimicrobial [1], anti‐inflammatory [2], antiviral [3], antioxidant [4], antipsychotic [5], antidepressant [6], antihistaminic [7], and antihypertensive [8]. Also, the anticancer activity of thiophene derivatives has been extensively documented [9, 10]. Thiophene is also a core structure in many marketed drugs, such as the antimigraine pizotifen, antihistamine ketotifen, antiplatelet ticlopidine, local anesthetic articaine, anticancer raltitrexed, hypnotic sedative indiplon, antiasthmatic zileuton, and antifungal tioconazole (Figure 1).

Pyrazole derivatives exhibit several biological effects, including antimicrobial, anticancer, analgesic, anti‐inflammatory, antiviral, antioxidant, antidiabetic, and neuroprotective activities [11]. These effects stem from the pyrazole moiety's ability to interact with a wide range of biological targets, creating a versatile lead for numerous medications, such as the analgesic metamizole, the anticoagulant apixaban, and the anti‐inflammatory celecoxib [12], in addition to the anticancer drugs crizotinib, pralsetinib, avapritinib, and asciminib [13]. Furthermore, recent research has confirmed that several thiophene–pyrazole hybrids show notable anticancer effects against the HepG2, MCF‐7, Ca9−22, and HSC‐2 cell lines [14, 15]. Motivated by these facts and as a continuation of our ongoing research on the synthesis of new chemical skeletons containing two or more bioactive moieties [16, 17, 18]. In this study, we report a convenient synthetic method for the synthesis of some new thiophene–pyrazole conjugates. The antitumor activity of the synthesized compounds against three tumor cell lines (HepG2, MCF‐7, and HCT‐116) was also presented.

## 2 Materials and Methods

### 2.1 Chemistry

#### 2.1.1 Instruments

The supplementary file provides additional details on all analytical and spectroscopic instruments used to characterize the synthesized compounds.

#### 2.1.2 Materials and Purification

All solvents and reagents were purchased from commercial sources, including Aldrich (purity >99%, St. Louis, MO, USA), and used without further purification. The remaining chemicals used in the work were of analytical reagent grade. Thiophene derivative **1** was prepared according to a previously reported method [19].

#### 2.1.3 Synthetic Procedures

##### 2.1.3.1 Synthesis of Ethyl 5‐(3‐(Dimethylamino)‐2‐(Ethoxycarbonyl)Acryloyl)‐4‐Methyl‐2‐(Phenylamino)Thiophene‐3‐Carboxylate (3)

A mixture of ethyl 5‐(3‐ethoxy‐3‐oxopropanoyl)‐4‐methyl‐2‐(phenylamino) thiophene‐3‐carboxylate (**1**) (0.75 g, 2 mmol) and *N*,*N*‐dimethylformamide dimethyl acetal (DMF‐DMA) (0.95 g, 1.06 mL, 8 mmol) was heated at 120°C for 30 min. After cooling to room temperature, the solid was filtered and recrystallized from an EtOH/DMF mixture to yield yellow crystals of the thiophene derivative **3** with an 80% yield, m.p. 141°C; IR (KBr) *ν* 3125 (NH), 1700, 1675 (2C=O), 1590 (C=C) cm^−1^; ^1^H NMR (400 MHz, DMSO‐*d*_6_) *δ* 1.04–1.07 (t, 3H, CH_3_, *J* = 5.7 Hz), 1.31–1.34 (t, 3H, CH_3_, *J* = 5.6 Hz), 2.56 (s, 3H, CH_3_), 2.76−3.11 (br., 6H, 2CH_3_), 3.97−4.01 (q, 2H, CH_2_, *J* = 5.7 Hz), 4.29−4.33 (q, 2H, CH_2_, *J* = 5.6 Hz), 7.18−7.47 (m, 5H, ArH), 7.51 (s, 1H, CH), 10.25 (s, 1H, NH); ^13^C NMR (100 MHz, DMSO‐*d*_6_) *δ* 14.6, 14.8, 16.3, 40.3, 40.4, 59.4, 60.7, 101.0, 110.0, 121.1, 125.2, 130.2, 140.4, 143.2, 153.5, 162.7, 166.0, 167.4, 185.0; MS (ESI–QTOF): calcd for [M + H]^+^ *m*/*z* = 431.16; found 431.1267. Anal. calcd. for C_22_H_26_N_2_O_5_S (430.52): C, 61.38; H, 6.09; N, 6.51; found: C, 61.25; H, 6.23; N, 6.38.

##### 2.1.3.2 Synthesis of Thiophene Pyrazole Hybrids 8a,b

##### 2.1.3.3 General Method

A mixture of thiophene derivative 3 (0.861 g, 2 mmol) and phenyl hydrazine or hydrazine hydrate (2.2 mmol each) in EtOH (10 mL) was heated under reflux for 3 h. The precipitated solid was collected by filtration and recrystallized from EtOH/DMF to afford the corresponding products **8a** and **8b**.

##### 2.1.3.4 Ethyl 5‐(4‐(Ethoxycarbonyl)‐3‐Methyl‐5‐(Phenylamino) Thiophen‐2‐yl)‐1‐Phenyl‐1H‐Pyrazole‐4‐Carboxylate (8a)

Yellow crystals, yield (70%); m.p. 156°C; IR (KBr) *ν* 1711, 1657 (2C=O), 1551 (C=C) cm^−1^; ^1^H NMR (400 MHz, DMSO‐*d*_6_) *δ* 1.18–1.21 (t, 3H, CH_3_, *J* = 5.7 Hz), 1.26–1.29 (t, 3H, CH_3_, *J* = 5.7 Hz), 1.91 (s, 3H, CH_3_), 4.15–4.20 (q, 2H, CH_2_, *J* = 5.7 Hz), 4.24–4.28 (q, 2H, CH_2_, *J* = 5.7 Hz), 7.09–7.48 (m, 10H, ArH), 8.23 (s, 1H, CH), 10.03 (s, 1H, NH); ^13^C NMR (100 MHz, DMSO‐*d*_6_) *δ* 14.5, 14.6, 16.6 (CH_3_), 60.3, 60.5 (CH_2_), 119.7, 124.2, 125.1, 129.0, 129.7, 130.1, 142.5 (CH's), 105.2, 107.8, 116.3, 137.6, 138.2, 139.3, 140.8, 160.6, 162.1, 165.6 (C's); MS (ESI–QTOF): calcd for [M + H]^+^ *m*/*z* = 467.16; found 476.1245; Anal. calcd. for C_26_H_25_N_3_O_4_S (475.56): C, 65.67; H, 5.30; N, 8.84; found: C, 65.82; H, 5.46; N, 8.77.

##### 2.1.3.5 Ethyl 5‐(4‐(Ethoxycarbonyl)‐3‐Methyl‐5‐(Phenylamino)thiophen‐2‐Yl)‐1H‐Pyrazole‐4‐Carboxylate (8b)

White crystals, yield (55%); m.p. 124°C; IR (KBr) *ν* 3346, 3250 (2NH), 1692, 1647 (2C=O) cm^−1^; ^1^H NMR (400 MHz, DMSO‐*d*_6_): *δ* 1.18–1.21 (t, 3H, CH_3_, *J* = 5.7 Hz), 1.32–1.34 (t, 3H, CH_3_, *J* = 5.7 Hz), 2.17 (s, 3H, CH_3_), 4.13–4.17 (q, 2H, CH_2_, *J* = 5.7 Hz), 4.30–4.34 (q, 2H, CH_2_, *J* = 5.7 Hz), 7.09–7.42 (m, 6H, ArH + NH), 8.23 (s, 1H, CH), 10.07 (s, 1H, NH); ^13^C NMR (100 MHz, DMSO‐*d*_6_): *δ* 14.6, 14.7, 16.9 (CH_3_), 60.0, 60.4 (CH_2_), 119.5, 123.9, 130.1, 137.5 (CH's), 108.4, 109.0, 112.9, 135.8, 141.1, 142.0, 159.0, 162.7, 166.0 (C's); MS (ESI–QTOF): calcd for [M + H]^+^ *m*/*z* = 400.13; found 400.0077. Anal. calcd. for C_20_H_21_N_3_O_4_S (399.47): C, 60.14; H, 5.30; N, 10.52; found: C, 60.32; H, 5.14; N, 10.44.

### 2.2 X‐Ray Crystallography

An ethanolic solution of compound **8a** was prepared and left to evaporate at room temperature, producing single crystals with a yellow block shape. A selected crystal was mounted on an Oxford Diffraction SuperNova diffractometer equipped with a HyPix3000 detector, and the intensity data were collected at 293 K using Mo K*α* radiation (*λ *= 0.71073 Å). Indexing of reflections, unit cell refinement, data collection, and numerical integration were carried out with the CrysAlis PRO software package. The structure solution was solved by means of SHELXT and refined by full‐matrix least‐squares techniques on F^2^ using SHELXL [20, 21], which are widely used for small molecule structure solution and refinement. All nonhydrogen atoms were refined with anisotropic displacement parameters, whereas hydrogen atoms were included in calculated positions and treated with a riding model. Molecular graphics and geometrical calculations were conducted using the Mercury 4.2.0 program [22]. The crystallographic data for **8a** can be obtained free of charge from the Cambridge Crystallographic Data Centre under deposition number 2527582 via www.ccdc.cam.ac.uk/data_request/cif.

### 2.3 Computational Analysis

#### 2.3.1 Hirshfeld Surface Analysis

Hirshfeld surface calculations [23] were carried out on the single‐crystal structure of **8a** in order to examine the intermolecular contacts in its crystal packing. The crystallographic information file of this compound was employed in CrystalExplorer 17.5 program [24] to generate Hirshfeld surfaces as well as the associated two‐dimensional fingerprint plots [25].

#### 2.3.2 Frontier Molecular Orbital Analysis and Global Reactivity Descriptors

**FIGURE 2:**
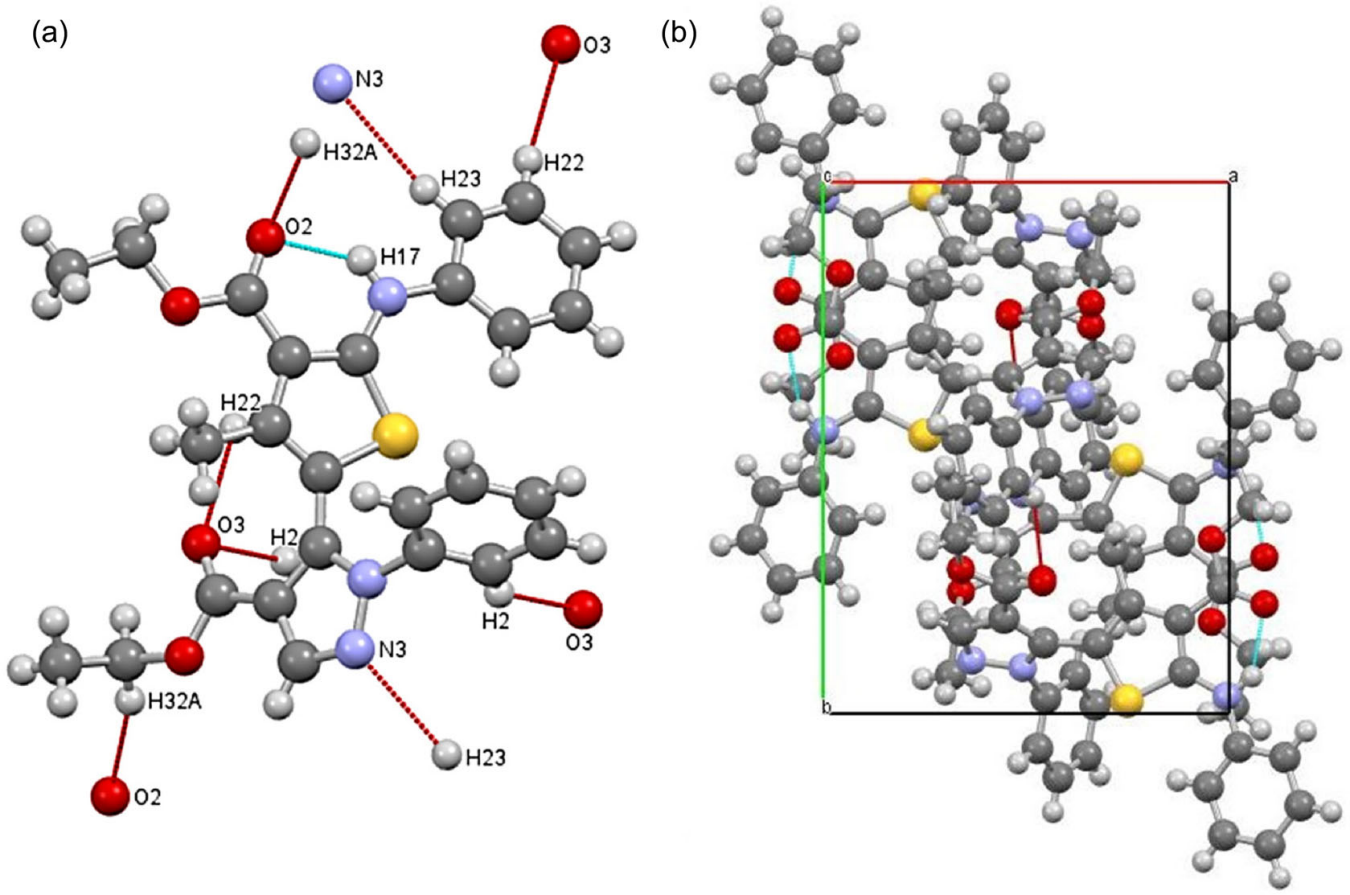
Intramolecular (turquoise) and intermolecular (red) interactions for compound **8a** in the crystal (a). Packing of compound **8a** in the unit cell along the *c*‐plane (b).

The chemical behavior of compound **8a**, such as the chemical reactivity, active sites, and kinetic stability, was studied using frontier molecular orbital calculations (FMO). FMO was calculated on the optimized structure of compound **8a** (Figure 2b), which was optimized at the density functional theory (DFT) level using the B3LYP method and basis sets of 6‐311++G(d, p) under neutral conditions [26] using Gaussian 09W program package [26]. The energies of the highest occupied molecular orbital (HOMO) and the lowest unoccupied molecular orbital (LUMO) were obtained from the quantum chemical output, and their spatial distribution was visualized using Gauss View 5.0. The HOMO is associated with electron‐donating ability, and the LUMO reflects the ability to accept electrons, and the pattern and localization of these orbitals indicate the potential reactive sites and charge transfer pathways within the molecule. The HOMO–LUMO energy difference (Δ*E*) is commonly used as an indicator of molecule's kinetic stability and chemical softness, where the large gaps are usually correlated with lower reactivity and greater thermodynamic stability [27].

In addition, the computed FMO energies were used to calculate the global reactivity descriptors for compound **8a** to obtain a more quantitative picture of the electronic structure of this compound. The descriptors include the ionization potential (*I*) and the electron affinity (*A*), electronegativity (*χ*), chemical potential (*μ*), global hardness (*η*), softness (*S*), which provide additional measures of the tendency of the molecule to donate or accept electrons, and its ability to act as an electrophile in the interactions with biological or chemical systems [28].

#### 2.3.3 Molecular Electrostatic Potential Mapping

The fully optimized geometry of the target compound was used to construct its molecular electrostatic potential (MEP), in order to examine the charge distribution over this molecule and to identify the regions that may be preferred for electrophilic and nucleophilic attacks. The resulting potential is presented using a color scale in which the electron‐rich sites (negative potential) are depicted in red or orange, while the electron‐deficient sites (positive potential) are typically shown in blue, indicating the preferred sites for electrophilic and nucleophilic attacks, respectively [29]. In addition, MEP surfaces are widely used to predict the availability and strength of the noncovalent interactions.

#### 2.3.4 Natural Bond Orbital and Mulliken Charge Analysis

The atomic charge distribution in compound **8a** was evaluated at the same DFT level of theory previously described using natural bond orbital (NBO) and Mulliken population analyses. NBO approach provides detailed information about the partitioning of the electron density among bonds and lone pairs. It is widely used to interpret structure, bonding, and reactivity in organic molecules [30]. Mulliken population analysis was also used to estimate the electronic charge distribution across the molecule. These analyses provide a complementary view of the electronic charge distributions within the target compound, indicating the electron‐rich and electron‐poor centers that may be relevant for intra‐ and intermolecular interactions.

### 2.4 In Silico Evaluation of Toxicity and Drug Likeness

The potential of compound **8a** to act as a drug‐like candidate was studied in silico by estimating the molecule's basic pharmacokinetic and toxicity‐related properties using the OSIRIS Property Explorer [31]. The program compares **8a**'s molecular structures with the known bioactive and toxic compounds using the compound's lipophilicity (log P), solubility (log S), molecular weight, toxicity risks, including mutagenicity, tumorigenicity, irritating effects, and reproductive effects.

### 2.5 The In Vitro Antitumor Assessment

The sulforhodamine B (SRB) assay was used to assess the anticancer efficacy of the synthesized compounds against the HepG2, MCF‐7, and HCT‐116 cell lines [32] [more details in the Supporting Information (SI) file].

## 3 Results and Discussion

### 3.1 The Synthesis of Thiophene Pyrazole Hybrids 8a,b

**SCHEME 1:**
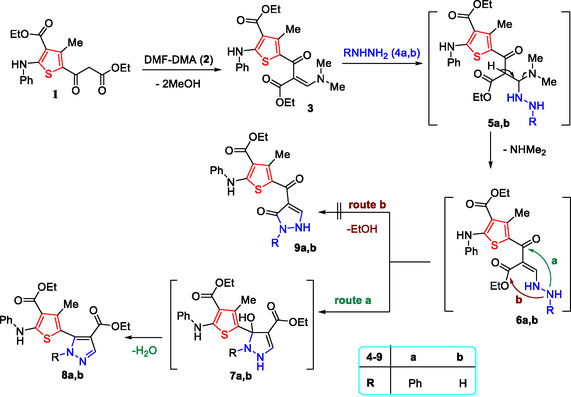
Synthesis of thiophene derivatives **3** and thiophene pyrazole conjugates **8a,b**.

The synthesis of thiophene pyrazole hybrids **8a,b**, as shown in Scheme 1, proceeded through two‐step reactions. In the first step, thiophene **1** [19] reacted with DMF‐DMA (**2**) to afford enaminone derivative **3**. The reaction of **3** with phenyl hydrazine (**4a**) or hydrazine hydrate (**4b**) afforded the target thiophene pyrazole hybrids **8a** and **8b**, respectively (Scheme 1).

The synthesis of **8a** and **8b** was proposed to proceed via initial addition of the NH_2_ group of hydrazine derivative **4a** or **4b** to the activated double bond in **3**, followed by loss of dimethylamine to give **6a,b**. These intermediates then underwent intramolecular cyclization and aromatization via loss of a water molecule (route a) to yield the target compounds **8a,b** (Scheme 1).

Both elemental and spectral techniques (infrared (IR), nuclear magnetic resonance (NMR), and mass spectroscopy (MS)) were used to confirm the chemical structures of the synthesized compounds.

For example, the NMR spectrum of **3** (Figures S2 and S3) showed the absence of signals at *δ* 3.77 and 48.73 ppm, corresponding to the proton and carbon of the ketonic CH_2_ group present in the spectrum of thiophene **1** [19]. Furthermore, its ^1^H NMR spectrum exhibited protons of two methyl groups from the dimethylamino moiety, as well as a singlet signal for the olefinic proton at *δ* 7.51 ppm.

The ^1^H NMR spectrum of **8a** showed all expected signals, including protons of the two ester groups at *δ* 1.18–1.29 and 4.15–4.28 ppm, as well as three singlets at *δ* 1.91, 8.23, and 10.03 ppm, corresponding to CH_3_, pyrazole‐H, and NH protons. Ten phenyl protons appeared as a multiplet in the 7.09–7.48 ppm region.

The ^13^C NMR spectrum of **8a** (Figures S6 and S7) showed signals at *δ* 14.5, 14.6, and 16.6 for three CH_3_; at *δ* 60.3 and 60.5 for two CH_2_; at δ 119.7, 124.2, 125.1, 129.0, 129.7, 130.1, and 142.5 for seven CH; and at *δ* 105.2, 107.8, 116.3, 137.6, 138.2, 139.3, 140.8, 160.6, 162.1, and 165.6 for 10 quaternary carbons. Finally, both mass and single‐crystal XRD data provided additional evidence for the assigned structure of compound **8a**.

Also, the proposed chemical structure of compound **8b** is consistent with its spectral data. For example, the ^13^C NMR spectrum of **8b** displayed signals at *δ* 14.6, 14.7, 16.9 for three CH_3_, at *δ* 60.0, 60.4 for two CH_2_, at *δ* 119.5, 123.9, 130.1, 137.5 due to four CH, and at *δ* 108.4, 109.0, 112.9, 135.8, 141.1, 142.0, 159.0, 162.7, 166.0 attributed to nine quaternary carbons.

### 3.2 X‐Ray Crystallography

**FIGURE 3:**
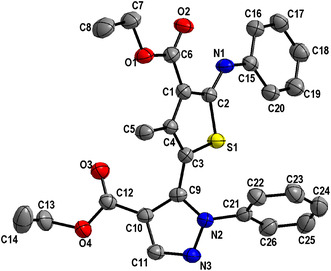
X‐ray structure of compound **8a**.

The proposed structure of compound **8a** was confirmed by single‐crystal X‐ray diffraction (Figure 3). The crystallographic data for this compound are shown in Table S1. The molecule's single crystal reveals a monoclinic lattice with space group P21/c and four molecules in the cell unit (*Z* = 4) as shown in Figure 2. The unit cell dimensions were found to be *a* = 11.3765(4) Å, *b* = 14.1839(5) Å, *c* = 15.3345(7) Å, with *α* = 79.723(4)°, forming a unit cell volume of 2357.55(17) Å^3^. The refinement finally converged at R1 = 0.0853 and wR2 = 0.2244, with a goodness‐of‐fit (*S*) of 1.041 as shown in Table S1.

Bond lengths and angles in the thiophene and pyrazole rings and ester groups (Table 1) fall within the normal ranges reported for related pyrazole–thiophene derivatives, confirming aromatic character and effective conjugation [33]. For example, the C—S distances (S1–C2 = 1.722(3) Å, S1–C3 = 1.740(3) Å) and the N—N distance (N2–N3 = 1.370(3) Å) (Table 1) are consistent with the bond lengths in aromatic thiophane and pyrazole fragments [34]. Both ethyl ester groups display short C=O bonds (C6–O2 = 1.219(4) Å, C12–O3 = 1.203(4) Å) and longer C—O (alkoxy) bond (C6–O1 = 1.341(4) Å, C12–O4 = 1.334(4) Å), consistent with the typical ester parameters [34] and indicating effective conjugation between the ester functions and the adjacent heteroaromatic scaffold.

**TABLE 1:** Selected bond lengths [Å] and angles [°] for compound 8a.

Bond	d, Å	Bond	d, Å
S1—C2	1.722(3)	N1—C2	1.349(4)
S1—C3	1.740(3)	N1—C15	1.411(4)
C3—C4	1.353(5)	O1—C6	1.341(4)
C4—C1	1.436(4)	O1—C7	1.435(4)
C1—C2	1.404(5)	O2—C6	1.219(4)
N2—N3	1.370(3)	O3—C12	1.203(4)
N2—C9	1.356(4)	O4—C12	1.334(4)
N3—C11	1.311(4)	O4—C13	1.444(4)
C9—C10	1.396(4)	C1—C6	1.455(5)
C10—C11	1.405(4)	C12—C10	1.463(4)

Angles	* θ*, °	Angles	* θ*, °
C2—S1—C3	91.63(16)	C3—C4—C5	122.8(3)
C9—N2—N3	112.6(3)	C1—C4—C5	125.1(3)
N3—N2—C21	117.8(2)	O2—C6—O1	121.8(3)
C9—N2—C21	129.6(3)	O2—C6—C1	125.3(3)
C11—N3—N2	104.8(2)	O1—C6—C1	113.0(3)
C2—N1—C15	132.7(3)	O3—C12—O4	123.6(3)
N2—C9—C10	105.2(3)	O3—C12—C10	125.8(3)
N2—C9—C3	122.6(3)	O4—C12—C10	110.6(3)
C10—C9—C3	132.2(3)	C26—C21—N2	119.4(3)
C3—C4—C1	112.0(3)	C16—C15—N1	116.3(3)

The molecule adopts a nonplanar conformation, with the aromatic rings twisted relative to one another. The thiophene and pyrazole ring planes are inclined nearly by 56°, and the *N*‐phenyl ring is coplanar with the thiophene (dihedral ≈ 13°), while the second phenyl group is more tilted (dihedral ≈ 69°–71° to the heteroaromatic rings).

The crystal packing of compound **8a** was stabilized by hydrogen bonding and weak contacts. The intramolecular N1—H17···O2 hydrogen bond (N1···O2 = 2.66 Å, N1—H17···O2 = 137.7°) stabilizes the generated six‐membered S(6) as shown in Table 2. In addition, the crystal packing is further supported by intermolecular weak and classical hydrogen bonds (Table 3). Short C—H···O and C—H···N interactions help linking the neighboring molecules, while C—H···*π* and other van der Waals contacts stabilize the three‐dimensional structure.

**TABLE 2:** Intramolecular hydrogen bond in compound 8a [Å and °].

D—H···A	d(D—H)	d(H···A)	d(D···A)	<(DHA)	Symm. Code
N1—H17···O2	0.860	1.96	2.66	137.7	*x*, *y*,*z*

**TABLE 3:** Selected weak intermolecular contacts in compound 8a [Å and °].

D—H···A	d(D—H)	d(H···A)	d(D···A)	<(DHA)	Symm. Code
C11—H9···O2	0.930	2.96	3.51	118.7	*x *− 1,*y*,*z*
C16—H23···N3	0.930	2.71	3.62	167.0	*x* + 1,*y*,*z*
C26—H2···O3	0.930	2.69	3.48	143.7	−*x* + 1,−*y* + 1,*z* + 1
C24—H4···O4	0.930	2.88	3.72	152.1	−*x* + 1,*y *‐ 1/2,*z* + 3/2

### 3.3 Hirshfeld Surface Analysis

**FIGURE 4:**
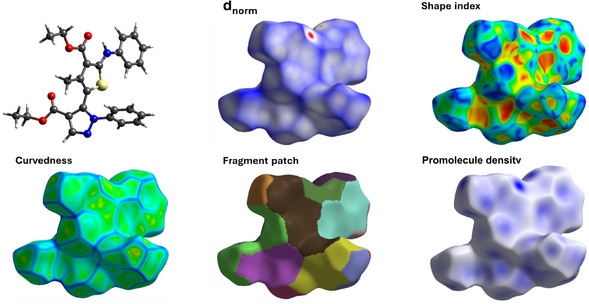
Hirshfeld maps for compound **8a**.

Hirshfeld surface analysis was conducted to study the short intermolecular contacts in the crystal packing of compound **8a**, based on the Hirshfeld partitioning scheme using CrystalExplorer. The three‐dimensional surface maps of compound **8a** are shown in Figure 4. The normalized contact distance (*d*_norm_) indicates the distance between a point on the Hirshfeld surface and the closest atoms of neighboring molecules, represented by a color from blue to red based on the distance. Red regions correspond to short intermolecular contacts shorter than the sum of van der Waals radii, the white regions represent distances close to van der Waals separation, while green and blue regions represent longer contacts. The intense red spots on the *d*_norm_ surface of **8a** are observed around the oxygen of the carbonyl group and around several nitrogen sites, including O···H and N···H, which identified crystallographically as hydrogen bond or short contacts. Most of the remaining surface is pale or blue as shown in Figure 4. The absence of the extended complementary red/blue triangles or large flat green patches in the shape index and curvedness maps (Figure 4) indicates that classical face‐to‐face *π*–*π* stacking is not dominant in this structure.

**FIGURE 5:**
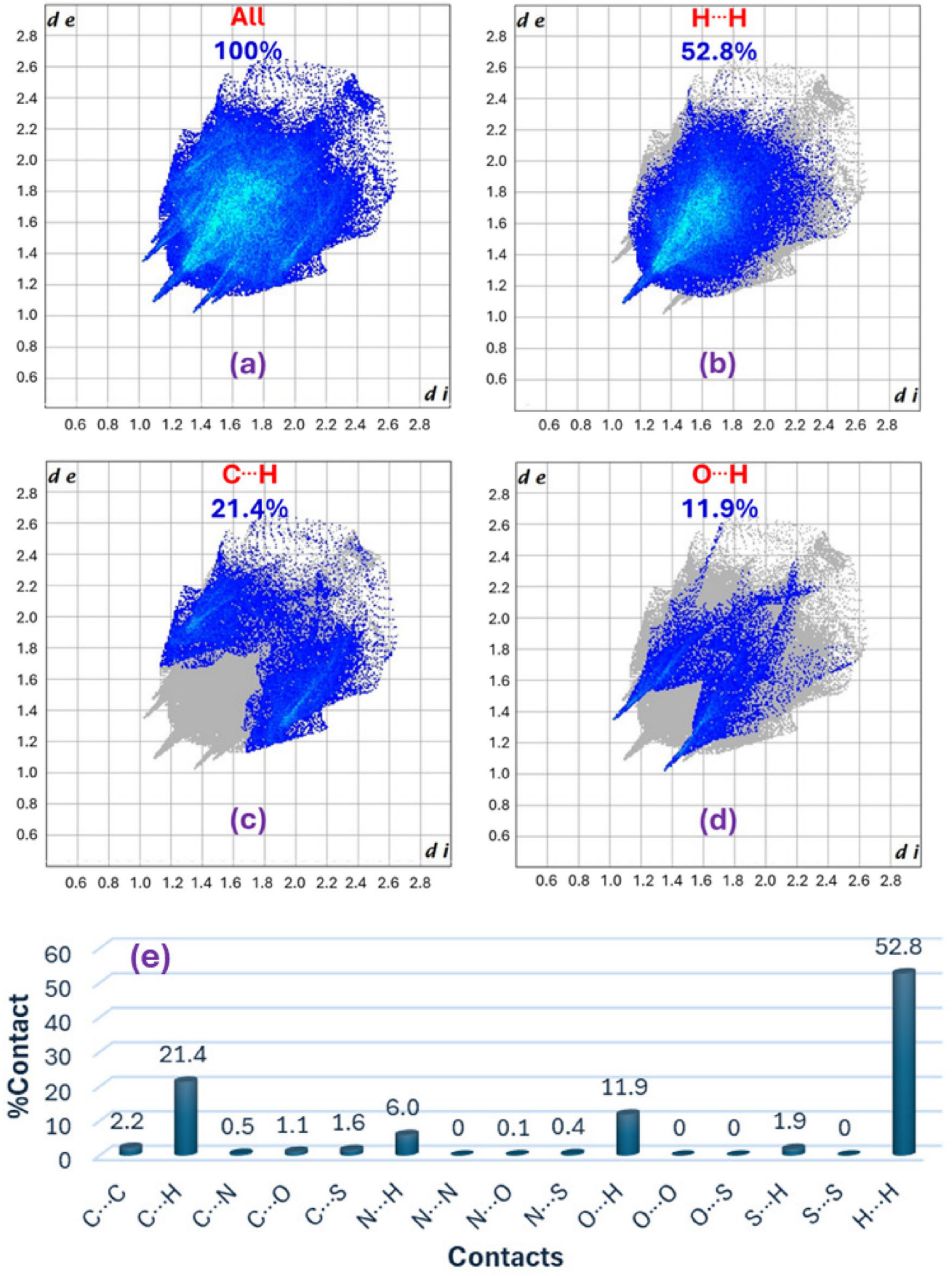
Decomposed fingerprint plots for the short contacts (a–d), and noncovalent interactions (e) in compound **8a**.

The intermolecular contacts were further studied using the two‐dimensional fingerprint plots (Figure 5). H···H contacts play an important role in the structural stability of **8a**, accounting for 52.8% associated with van der Waals interactions. Other dominated interactions include C···H/H···C (21.4%), O···H/H···O (11.9%), and N···H/H···N (6.0%). The O···H/H···O contribution is consistent with the hydrogen bonds in C—H···O and N—H···O which identified in the crystallographic analysis. The large contribution of C···H/H···C indicates the importance of hydrogen to carbon contacts in the packing. However, examining the H···centroid distances and C—H···centroid angles did not provide clear geometric evidence for a significant edge to face C—H···*π* interaction. In addition, the very small contributions from C···C (2.2%) and C···N/N···C (0.5%) indicates that the direct face‐to‐face *π–* *π* stacking is not a dominant feature in this structure. Therefore, the crystal packing of **8a** is stabilized mainly by weak hydrogen bonds and van der Waals contacts rather than significant aromatic stacking interactions.

### 3.4 Molecular Electrostatic Potential

**FIGURE 6:**
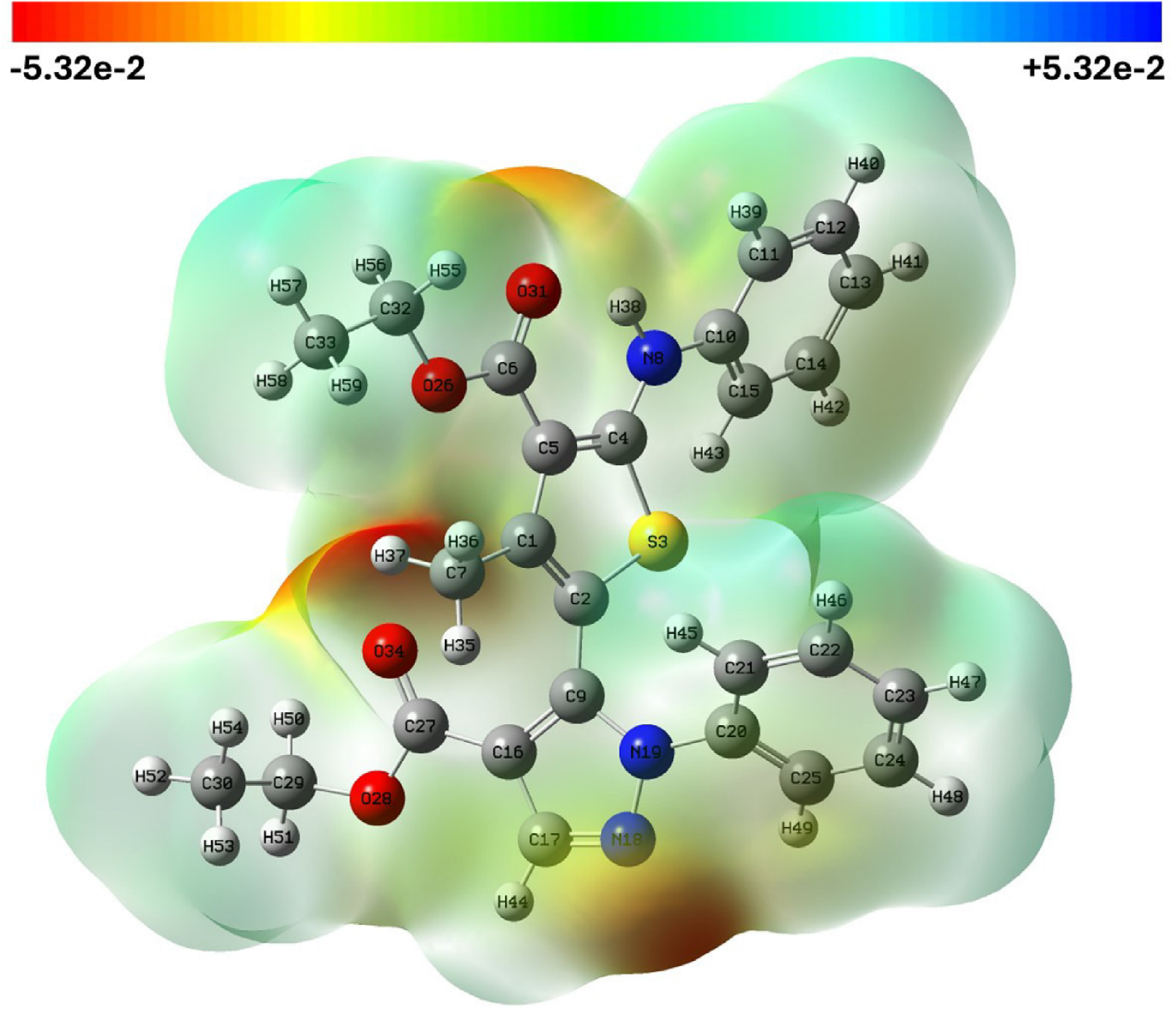
The MEP map for compound **8a**. MEP = Molecular electrostatic potential.

The MEP surface of compound **8a** was mapped to identify the preferred sites for electrophilic and nucleophilic interactions. The electrostatic potential is presented on the surface in different colors from red to blue and ranges between −5.32e‐2 and 5.32e‐2 a.u. for **8a** as shown in Figure 6. The red zones correspond to negative potential and electron‐rich zones, while the blue zones correspond to positive potential and electron‐deficient zones. Zones with green color correspond to nearly neutral regions.

In compound **8a**, carbonyl oxygens and the nitrogen of the pyrazole exhibit the most negative regions, indicating favorable sites for electrophilic attack. Therefore, these sites may act as the primary hydrogen bond acceptors consistent with the short C—H···O and N—H···O contacts which observed in the crystallographic analysis. In contrast, hydrogen atoms of the phenyl rings and ethyl groups exhibit the most positive potential, making them the most preferred as hydrogen bond donors or interacting with electron‐rich regions of biomolecular targets.

### 3.5 Frontier Molecular Orbital Insights

FMO is essential to study the chemical reactivity of an organic compound, as HOMO is associated with the tendency to donate electrons, and LUMO reflects the tendency to accept electrons. HOMO and LUMO energies are linked to the ionization potential and electron affinity, where IP ≈ −*E*_HOMO_ and EA ≈ −*E*_LUMO_. The difference between HOMO and LUMO energies is widely used as a measure of the molecule's chemical hardness and kinetic stability.

**FIGURE 7:**
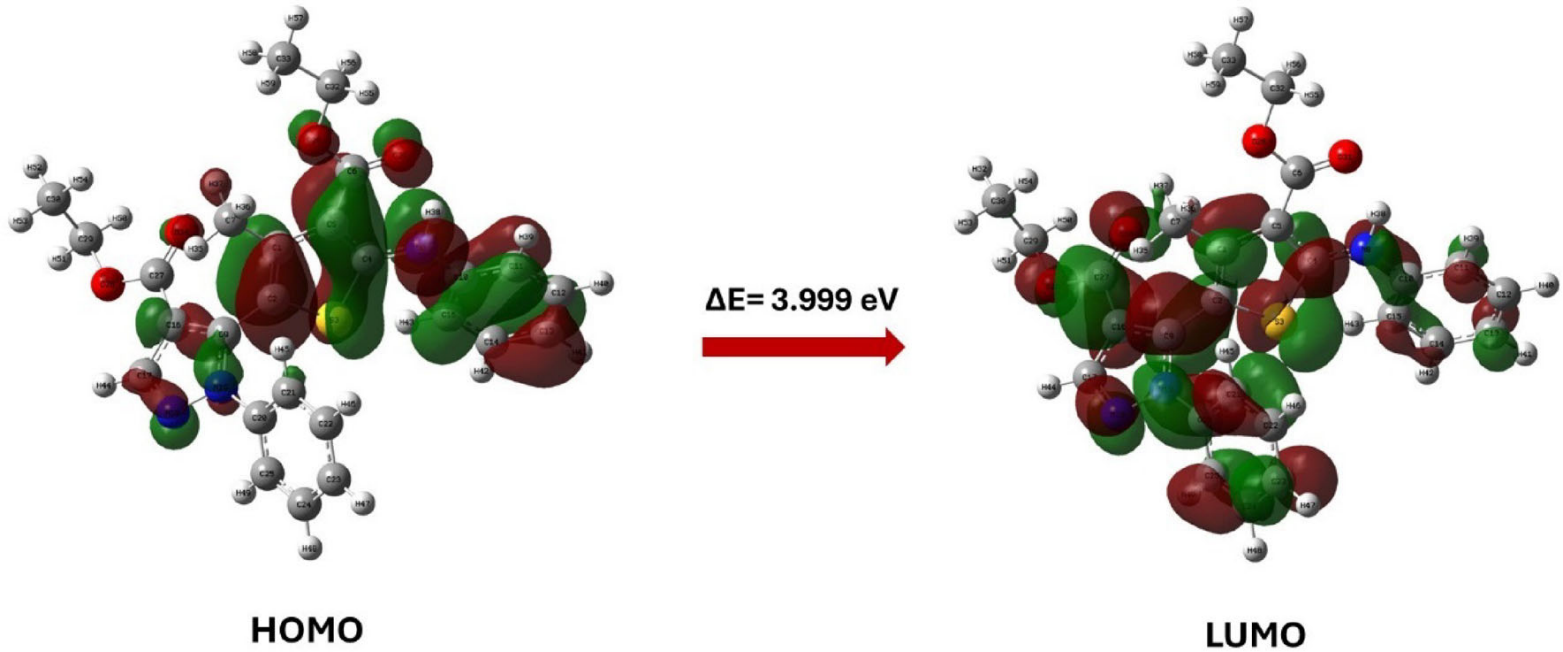
The HOMO and LUMO levels for compounds **8a**. HOMO = Highest occupied molecular orbital; LUMO = lowest unoccupied molecular orbital.

The obtained results showed that compound **8a** has HOMO of −5.124 eV and LUMO of −1.125 eV, giving an energy gap Δ*E* of 3.999 eV as shown in Figure 7 and Table 4. Energy gap of this magnitude suggests a molecule that is electronically stable but at the same time is a soft to participate in charge transfer processes. It has been reported that similar gaps have been associated with a balance between stability and reactivity in other heteroaromatic systems [35]. In addition, similar gaps are linked to moderate polarizability, which is relevant for both noncovalent recognition and potential optical responses [36].

**TABLE 4:** HOMO, LUMO, energy gap, ionization potential (IP), electron affinity (Ea), chemical potential (*μ*), chemical hardness (*η*), softness (*S*), and electronegativity (*χ*) of compound 8a in eV.

Reactivity parameters	Value, eV
HOMO	−5.124
LUMO	−1.125
Energy gap	3.999
Ionization potential (IP)	5.124
Electron affinity (Ea)	1.125
Chemical potential (μ)	−3.125
Chemical hardness (*η*)	1.999
Softness (*S*)	0.500
Electronegativity (*χ*)	3.125

Figure 7 shows that the HOMO is predominantly concentrated over the thiophene–pyrazole core and the adjacent phenyl ring, indicating that these regions are electron‐rich and able to donate electrons during interactions with electrophiles and electron poor sites in biomolecules. On the other hand, the LUMO extends over the ester carbonyl groups and nearby aromatic fragments, indicating the role of these systems to accept electrons.

The global reactivity descriptors derived from the HOMO and LUMO energies (Table 4) further clarify the electronic character of compound **8a** and calculated based on the following equations [37].

Hardness: $\eta =\frac{\left(\mathrm{IP}-\mathrm{Ea}\right)}{2}$

Chemical potential: $\mu =-\frac{\left(\mathrm{IP}+\mathrm{Ea}\right)}{2}$

Softness: $S=\frac{1}{\eta }$

Electronegativity: $\chi =\frac{\left(\mathrm{IP}+\mathrm{Ea}\right)}{2}$

The estimated values of these reactivity descriptors indicate a molecule of intermediate hardness, in line with the moderated HOMO–LUMO gap.

In conclusion, FMO analysis and reactivity descriptors indicate that the target compound has a stable conjugated framework with enough electronic flexibility to engage in both donor and acceptor interactions align with its proposed chemical and biological behaviors.

### 3.6 Mulliken Charge Analysis

**FIGURE 8:**
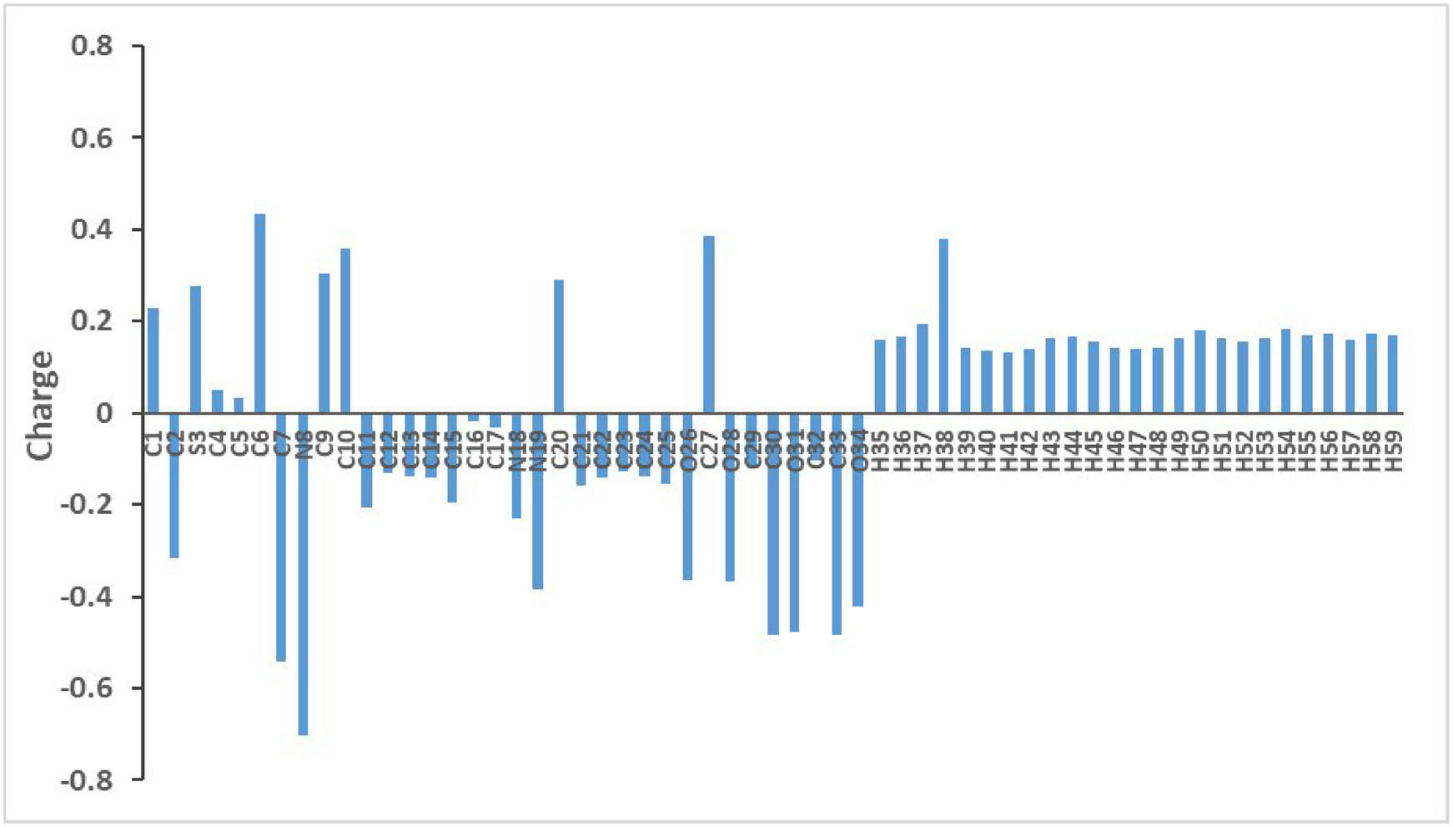
Plot of Mulliken charge for compound **8a**.

The electron density in compound **8a** was studied using Mulliken population analysis to identify its potential donor and acceptor sites. The calculated charges for compound **8a** are shown in Table 5 and Figure 8.

**TABLE 5:** Mulliken charges for compound 8a.

Atom	Charge	Atom	Charge
C1	0.22867	O31	−0.47604
C2	−0.31674	C32	−0.1051
S3	0.276541	C33	−0.48518
C4	0.051466	O34	−0.42322
C5	0.032403	H35	0.158778
C6	0.433025	H36	0.1654
C7	−0.54339	H37	0.192997
N8	−0.70299	H38	0.380207
C9	0.303184	H39	0.14274
C10	0.359478	H40	0.137606
C11	−0.20617	H41	0.133187
C12	−0.13076	H42	0.13903
C13	−0.13941	H43	0.164376
C14	−0.13977	H44	0.168189
C15	−0.19554	H45	0.157354
C16	−0.01822	H46	0.1416
C17	−0.03288	H47	0.138051
N18	−0.23148	H48	0.141703
N19	−0.38385	H49	0.163708
C20	0.289367	H50	0.180023
C21	−0.15919	H51	0.161693
C22	−0.14017	H52	0.157423
C23	−0.12833	H53	0.164605
C24	−0.13843	H54	0.182999
C25	−0.1549	H55	0.170488
O26	−0.36328	H56	0.173117
C27	0.387533	H57	0.161267
O28	−0.36883	H58	0.173176
C29	−0.11261	H59	0.168811
C30	−0.4837	—	—

As expected, oxygen and nitrogen atoms exhibit negative charges (pyrazole nitrogen N8 is the largest), indicating that these sites are the principal electron‐rich in the molecule; therefore, they likely participate as acceptors in hydrogen bonding or interact with electrophilic residues. Several aromatic carbons exhibit moderate negative charge while the phenyl carbons (C11–C15 and C21–C25) show small negative charges, indicating that the *π* system in the phenyl rings retains the electron density which can support the electron donating capacity. In addition, C7 and C30 show negative charge highlighting additional sites that can participate in weak intermolecular contacts.

On the other hand, carbon atoms directly connected to electronegative atoms (oxygen and nitrogen) carry positive charges (C6 has the highest charge). All hydrogen atoms carry positive charges. H38 bonded to the secondary amine nitrogen exhibits the largest value. These hydrogens represent potential hydrogen bond donors in agreement with MEP results and the observed hydrogen bonding in the crystal packing. Thiophene's sulfur (S3) also shows positive polarization which may be attributed to the overall withdrawal of electron density by the surrounding conjugated framework. This is consistent with our reported work [18].

### 3.7 In Silico Evaluation of Toxicity and Drug Likeness

The toxicity and drug likeness of compounds **3**, **8a**, and **8b** were evaluated in silico using the ORISIS Property Explorer interface. ORISIS tool enables an early stage prediction of the compound toxicity risks and physicochemical properties using color flags [31].

ORISIS shows green mutagenicity, tumorigenicity, irritancy, and reproductive effects in the case of **8a** and **8b**, as shown in Table 6, indicating that their structures passed the preliminary safety tests. Compound **3** also showed green flags for mutagenicity, tumorigenicity, and reproductive effects, but gave a medium risk irritancy alert. This warning arises from a fragments‐based structure in this molecule according to ORISIS. The molecular weights of **3**, **8a**, and **8b** (430.0, 475.0, and 399.0 g/mol, respectively) are within the Lipinski limit for oral drugs (MW < 500) [38]. These compounds also showed a topological polar surface area (TPSA) values of 113.1, 110.6, and 121.5 Å^2^, respectively, and below the Veber limit (140 Å^2^), indicating acceptable polarity for oral bioavailability [39]. Compound **8a** was the most lipophilic analogue (cLogP = 5.3) and is slightly higher than the Lipinski's rule of five threshold (5.0). High lipophilicity is often correlated with low aqueous solubility, so **8a** showed the lowest predicted solubility (log S = −6.0), whereas **3** and **4b** were less lipophilic, with cLogP = 3.45 and 4.03 values, respectively. The drug‐likeness values were −5.39 for **3**, 0.67 for **8a**, and −0.47 for **8b**, suggesting that **8a** structure motifs resemble those found in known bioactive molecules in OSIRIS reference set, while **3** and **8b** values suggest that some of their fragments are less common among the marketed drug structures. The calculated drug score values were 0.25, 0.30, and 0.41 for **3**, **8a**, and **8b**, respectively. **8b** showed the highest drug score and no predicted toxicity alerts, indicating the most balanced in silico profile, while compound **3** remains interesting because of its strong biological activity despite a less favorable in silico profile.

**TABLE 6:** The predicted physicochemical properties and toxicity risks of compounds 3, 8a, and 8b, generated by the OSIRIS Property Explorer interface.

Analysis	3	8a	8b
Mutagenicity	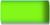	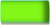	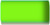
Tumorigenicity	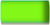	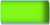	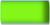
Irritancy	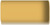	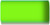	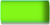
Reproductive effectiveness	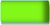	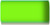	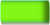
Molecular weight (MW, g mol^−1^)	430.0	475.0	399.0
Partition coefficient (cLogP)	3.45	5.3	4.03
Aqueous solubility (mol L^−1^)	−4.47	−6.0	−4.84
TPSA (Å^2^)	113.1	110.6	121.5
Drug likeness	−6.39	0.67	−0.47
Drug score (0–1)	0.25	0.30	0.41

### 3.8 Antitumor Evaluation

The anticancer properties of the synthesized compounds were evaluated against three human cancer cell lines, namely MCF‐7, HepG2, and HCT‐116, using the sulforhodamine B (SRB) assay [32].

As shown in Table 7, thiophene derivative **3** had significant inhibitory efficacy against MCF‐7 cancer cells, exhibiting an IC_50_ value of 2.2 μg/mL, compared to Doxorubicin's IC_50_ value of 0.92 μg/mL Also, thiophene pyrazole hybrid **8b** is more potent compared to **8a** against MCF‐7, HepG2, and HCT‐116 cancer cells.

**TABLE 7:** The cytotoxic activities of synthesized compounds on MCF‐7, HepG2, and HCT‐116 cancer cells.

Compound	IC_50_, μg/mL		
	MCF‐7	HepG2	HCT‐116
**3**	2.2 ± 0.3	27.2 ± 1.2	≥100
**8a**	≥100	≥100	>100
**8b**	8.9 ± 0.7	13.9 ± 1.9	10.02 ± 0.83
Doxorubicin (Dox)	0.92 ± 0.091	0.62 ± 0.04	1.2 ± 0.12

## 4 Conclusion

Thiophene derivative **3** was prepared and used as a precursor for the synthesize of two new thiophene‐appended pyrazoles, **8a** and **8b**. The structures of synthesized compounds were confirmed by IR, NMR, and MS, while compound **8a** was further characterized by single‐crystal X‐ray diffraction and computational analysis. The crystal structure of **8a** revealed a twisted structure stabilized by intermolecular N—H···O hydrogen bonding and by weak C—H···O and C—H···N contacts, together with many H···H and C···H interactions in the crystal packing. Hirshfeld surface analysis confirmed that H···H contacts are the main packing contribution, while the small C···C contribution indicated that direct face‐to‐face *π*···*π* stacking is not a significant feature of this structure. The computational study supported the structural findings and showed that **8a** possesses a moderate HOMO–LUMO gap (3.999 eV) indicating a conjugated system that is electronically stable but still able to participate in charge–transfer interactions. The antitumor activity of the synthesized compounds was evaluated against the HepG2, MCF‐7, and HCT‐116 cell lines. The IC_50_ assay demonstrated that thiophene derivative **3** showed promising activity against MCF‐7 cancer cells, with an IC_50_ of 2.2 μg/mL, compared with Doxorubicin's IC_50_ of 0.92 μg/mL. Moreover, thiophene pyrazole hybrid **8b** shows higher activity than **8a** against MCF‐7, HepG2, and HCT‐116 cancer cells. The superior performance of **8b** over **8a** may be attributed to the difference in substitution on the pyrazole–thiophene framework. The OSIRIS in silico safety and drug‐likeness evaluation showed that **8a** and **8b** have no predicted alerts for mutagenicity, tumorigenicity, irritancy, and reproductive effects, while compound **3** showed a medium irritancy alert. Overall, **8b** is the most promising compound, while compound **3** remains of interest because of its strong activity against MCF‐7 cancer cells. These findings support further optimization of thiophene–pyrazole hybrids as potential anticancer agents.

## Supporting Information

Additional SI can be found online in the Supporting Information section.

## Funding

This study was supported by Deanship of Scientific Research, King Khalid University (RGP2/733/46).

## Conflicts of Interest

The authors declare no conflicts of interest.

## Supporting information

Supplementary Material

## Data Availability

The data supporting this study's findings are available in this article's SI.
