# Supplementary material for: Synthesis, X‐Ray Analysis, Anticancer Activity, Computational, and in Silico Studies of New Thiophene Pyrazole Conjugates
Source: ChemistryOpen. 2026 May 18;15(6):e70226. doi: 10.1002/open.70226 (PMC13181806; doi:10.1002/open.70226)

Synthesis, X-ray analysis, anticancer activity, computational, and in silico studies of new thiophene pyrazole conjugates

Abdullatif Bin Muhsinah[a], Nabila A. Kheder[b], Ismail A. Elhaty[c,*], and Yahia N. Mabkhot[d,*]

Supporting Information

Table of contents **Pages**

1. Chemistry (General part)……………………………………………. **2**
2. The in vitro antitumor assessment …………………………………… **2**
3. Characterization data…………………………………………………… **3**
Fig. S1. Mass spectrum of compound **3**………………………………….. **3**
Fig. S2. ^{1}H NMR spectrum of thiophene **3** at 400 MHz in DMSO-d_6….. **4**
Fig. S3. ^{13}C NMR spectrum of **3** in DMSO-d_6 at 100 MHz ……………...... **4**
Fig. S4. Mass spectrum of compound **8a**………………………………. **5**
Fig. S5. ^{1}H NMR spectrum of **8a** at 400 MHz in DMSO-d_6……………… **5**
Fig. S6. ^{13}C NMR spectrum of **8a** at 100 MHz in DMSO-d_6…….……… **6**
Fig. S7. ^{13}C NMR- DEPT-135 spectrum of **8a**……………………….…... **6**
Fig. S8. Mass spectrum of compound **8b**………………………………. **7**
Fig. S9. ^{1}H NMR spectrum of **8b** at 400 MHz in DMSO-d_6……………… **7**
Fig. S10. ^{13}C NMR spectrum of **8b** at 100 MHz in DMSO-d_6…….……… **8**
Fig. S11. ^{13}C NMR- DEPT-135 spectrum of **8b**…………………………. **8**
Table S1. X-ray data of compound **8a**.…………………………………… **9**
4. CheckCIF data of compound 8a …………………………………….. **10**

1. Chemistry

General remarks

In open glass capillaries, all melting points were determined using a Gallen-Kamp apparatus (Thermo Fisher Scientific, Paisley, UK) and were uncorrected. Infrared (IR) spectra were measured using the potassium bromide disk technique on Perkin Elmer FT-IR 1000 (PerkinElmer, Waltham, MA, USA). A Bruker advanced NMR spectrometer running at 400 MHz in deuterated DMSO was used to measure the NMR spectra. Elemental composition was analyzed using a Vario Elemental Analyzer III (Vario, Germany). Using the sulforhodamine B assay, the anticancer

properties of the synthesized compound were assessed against three human cancer cell lines: MCF-7, HepG2, and HCT-116.

2. The in vitro antitumor assessment

Cell lines

Liver carcinoma cells (HepG2), Human breast adenocarcinoma (MCF-7), and colon adenocarcinoma (HCT 116) cell lines were provided by the American Type Culture Collection (ATCC, Boston, MA, USA). RPMI 1640 medium, supplemented with 10% foetal bovine serum and 100 IU/mL PS (penicillin/streptomycin), was used to culture HepG2, MCF-7, and HCT 116 cell lines

Cell viability assay

The sulforhodamine B (SRB) assay was used to assess the anticancer efficacy of compound **8**. HepG2, MCF-7, and HCT-116 cell lines were plated in 96-well plates at a density of 2×10^3 cells per well. The cells were left to adhere for a full day before being treated with nanoemulsion applications. Various concentrations of the formulations (0, 0.01, 0.1, 1, 10, 100, and 1000 μg/mL) were applied to the wells, and the cells were incubated for 72 hours at 37 °C. After this incubation period, the culture media were replaced with 150 μL of 10% trichloroacetic acid and incubated for 1 h. at 4 °C, followed by five washes with distilled H_2O. The cells were then stained with 50 μL of a 0.04% w/v SRB solution and incubated for 10 min. at 25 °C in the dark. Afterward, the excess stain was removed with 1% CH_3COOH, and 100 μL of 10 mM Tris base solution (pH=10.5) was added to each well to dissolve the protein-bound dye. The optical density was measured using a microplate reader set to 540 nm [32].

3. Characterization data

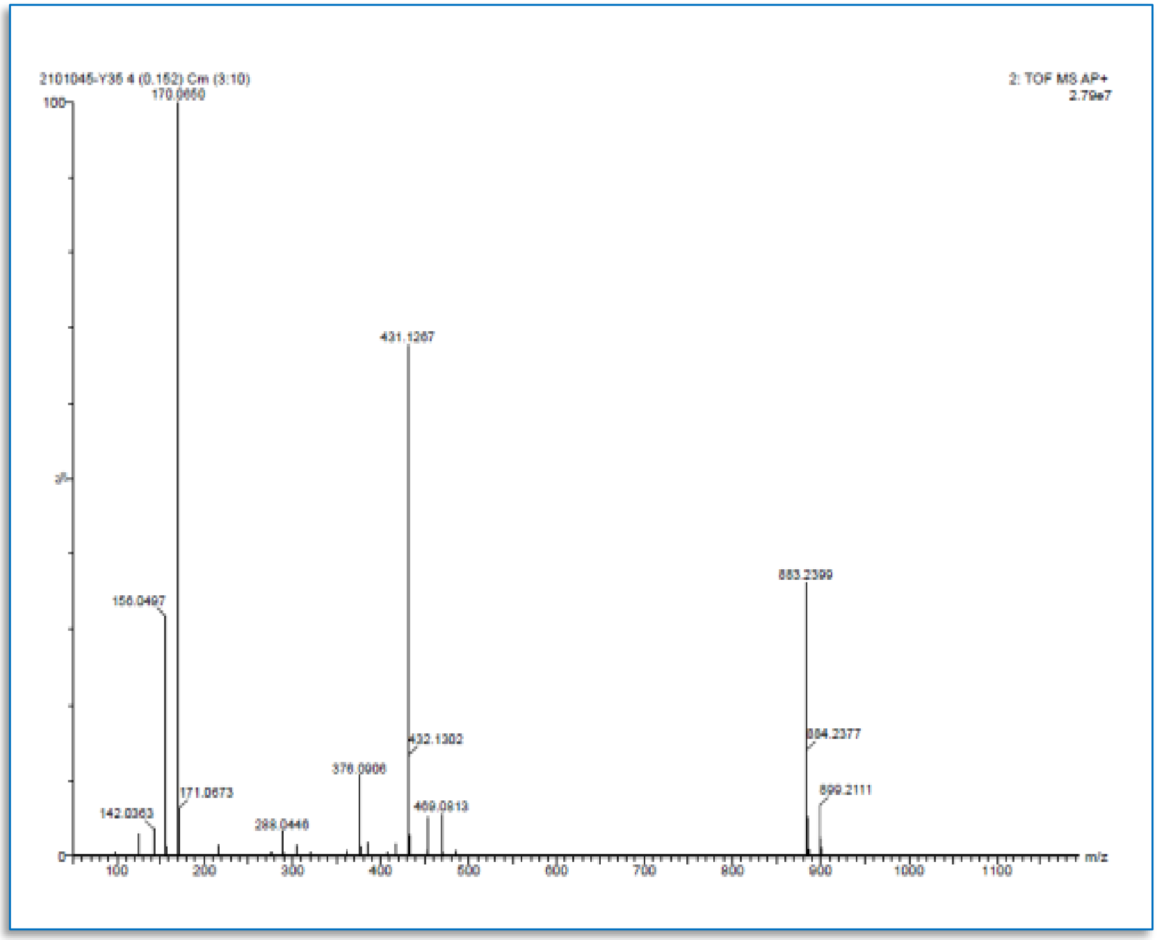

Fig. S1. Mass spectrum of compound **3**.

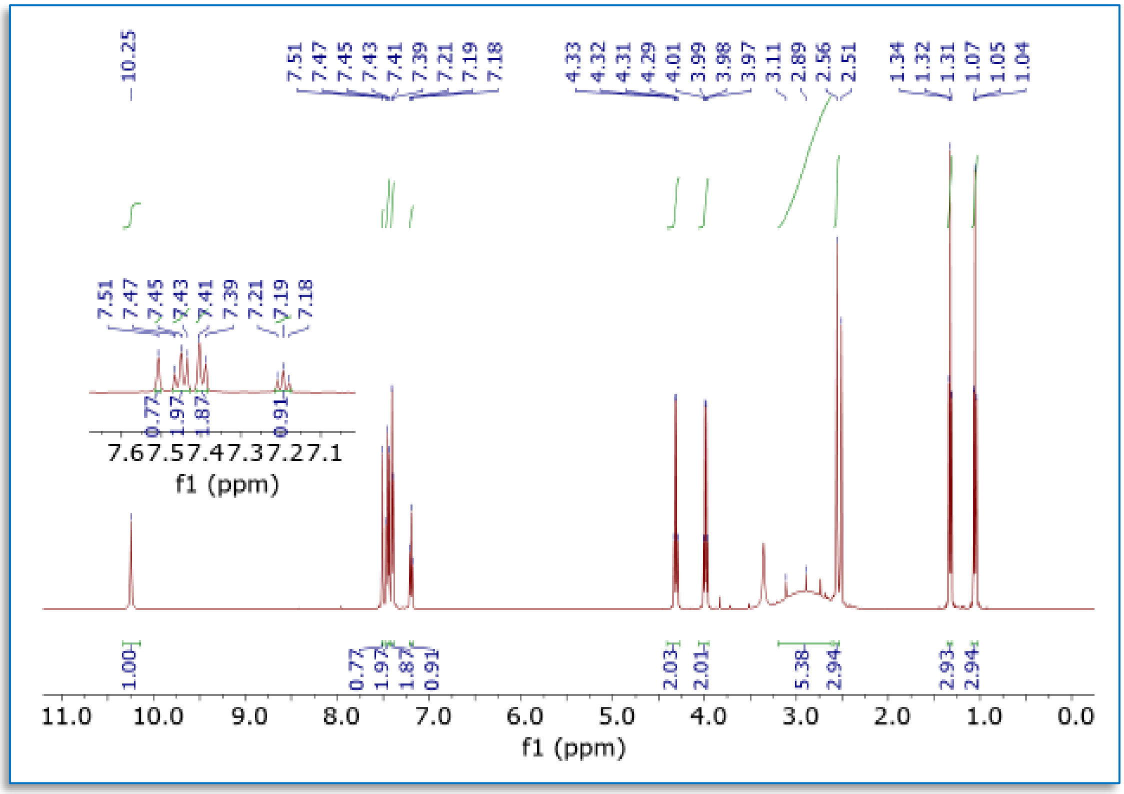

Fig. S2. ^{1}H NMR spectrum of thiophene **3** at 400 MHz in DMSO-d_6.

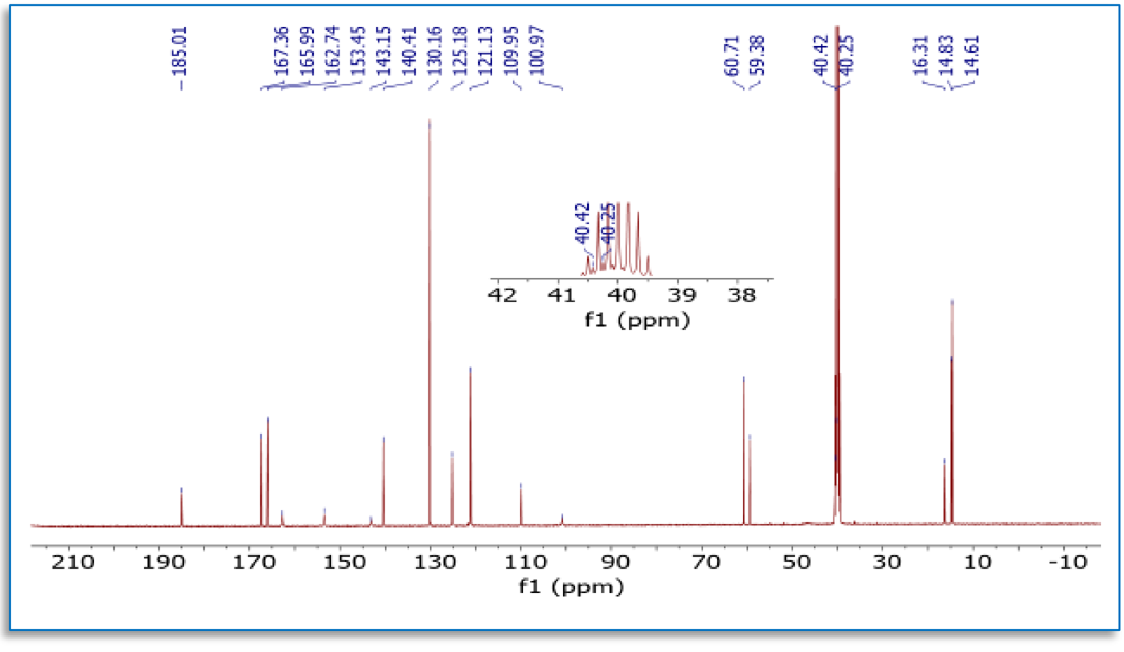

Fig. S3. ^{13}C NMR spectrum of **3** in DMSO-d_6 at 100 MHz.

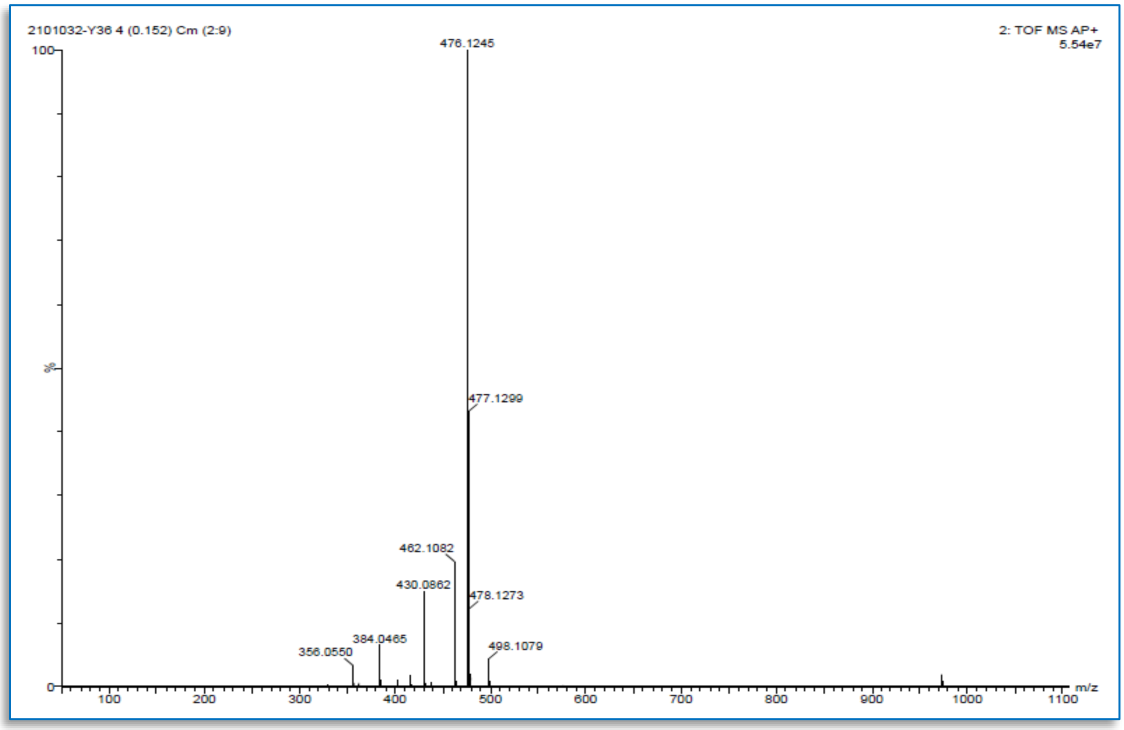

Fig. S4. Mass spectrum of compound **8a**.

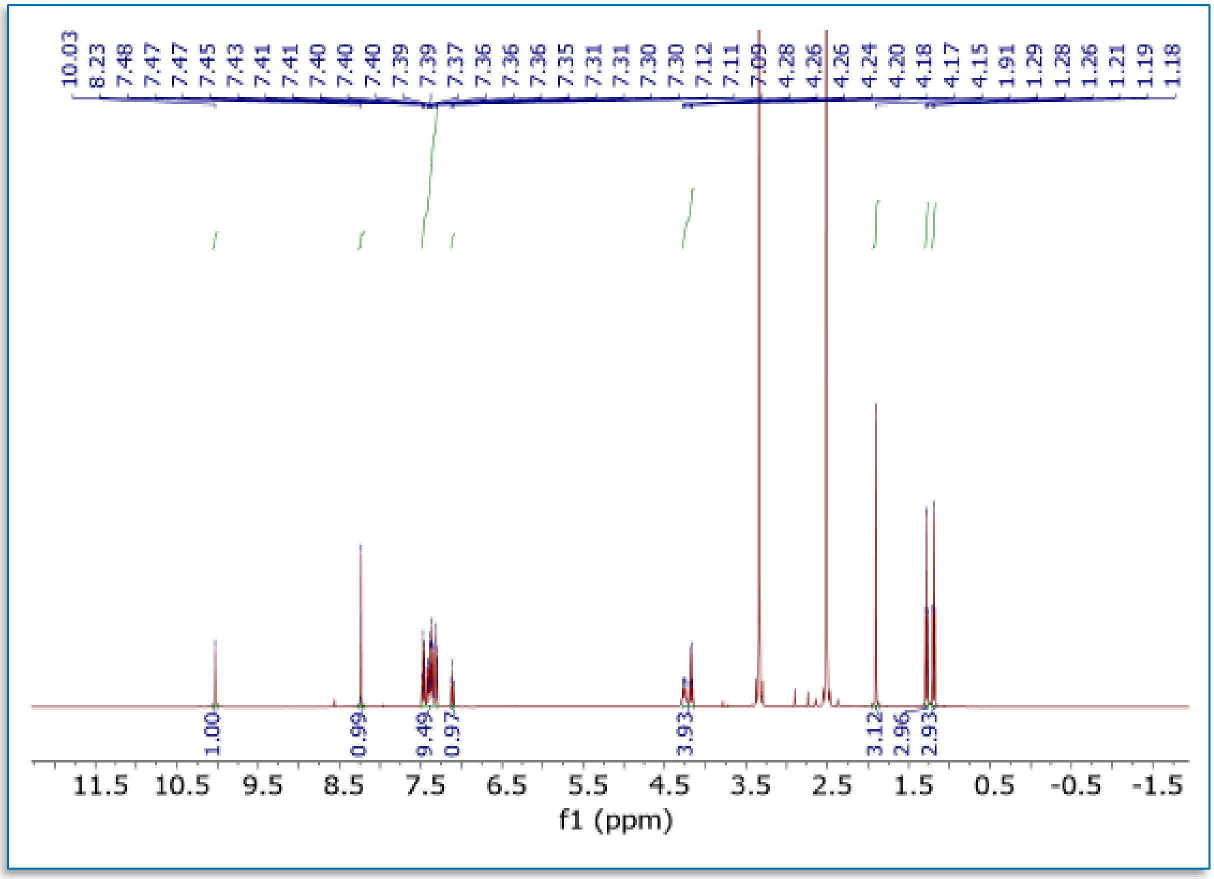

Fig. S5. ^{1}H NMR spectrum of **8a** at 400 MHz in DMSO-d_6.

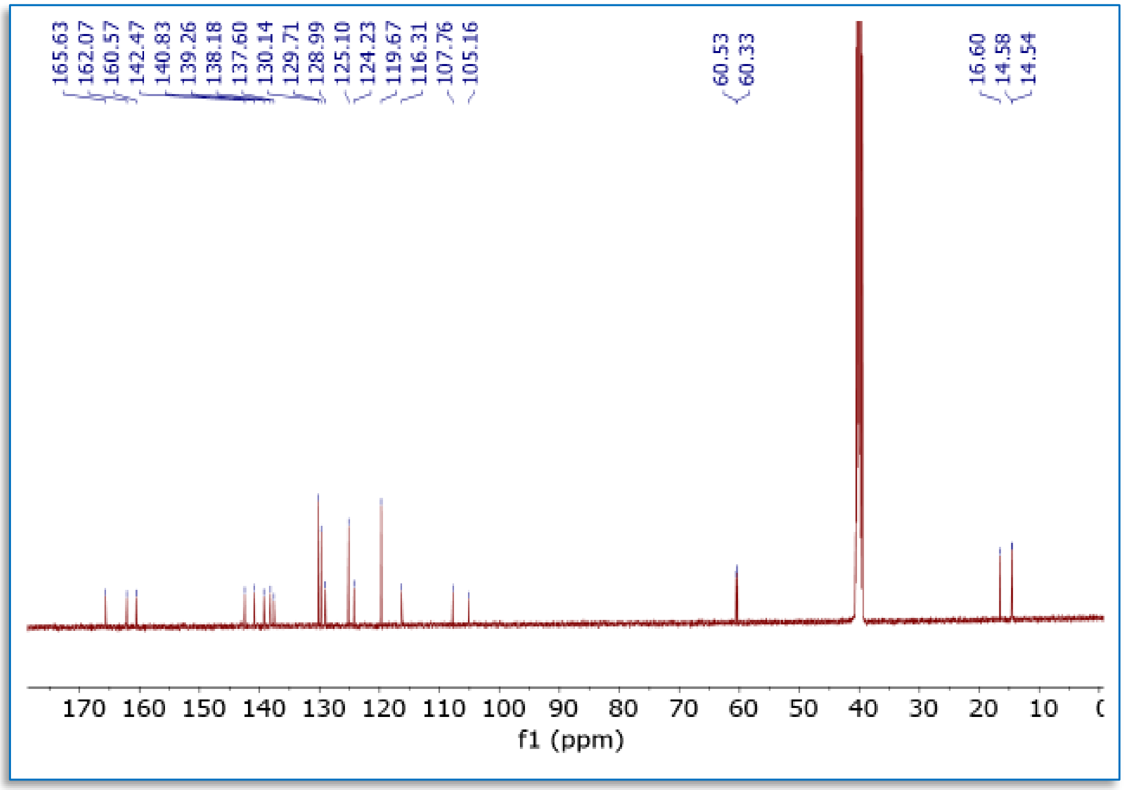

Fig. S6. ^{13}C NMR spectrum of **8a** at 100 MHz in DMSO-d_6

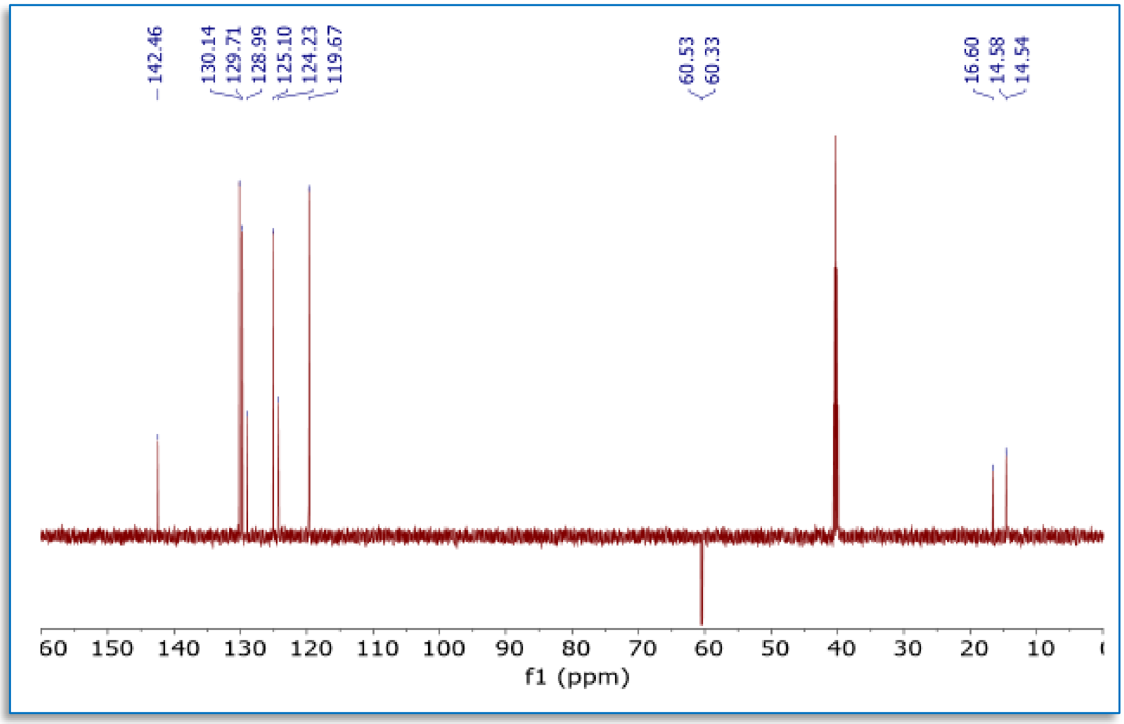

Fig. S7. ^{13}C NMR- DEPT-135 spectrum of **8a**.

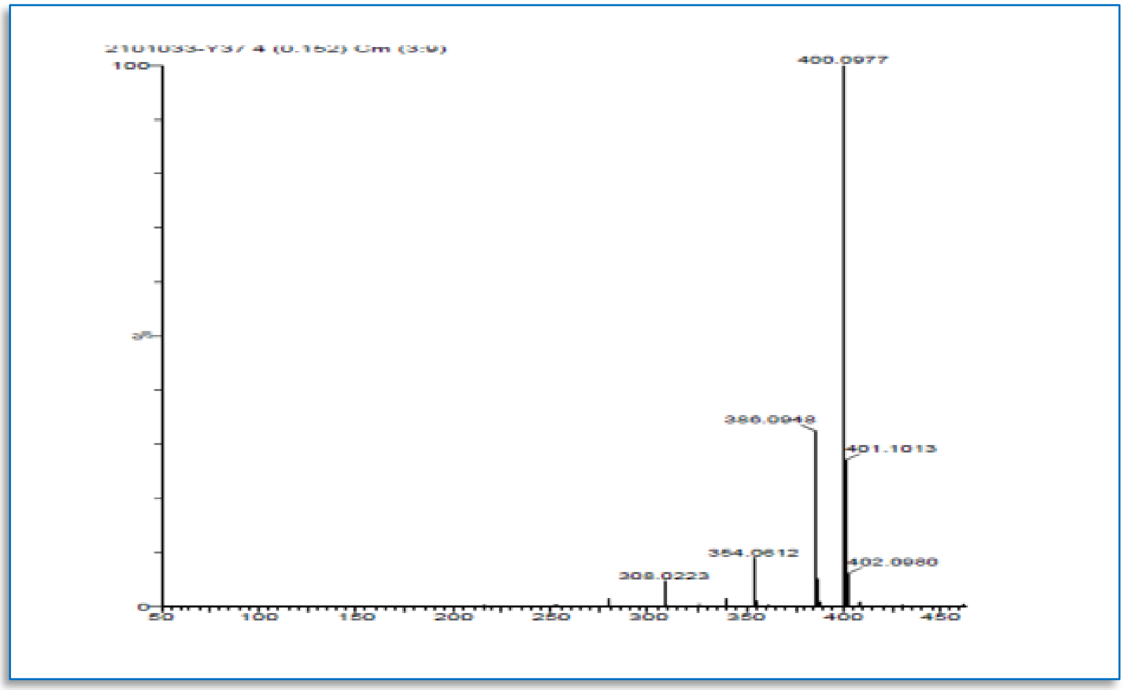

Fig. S8. Mass spectrum of compound **8b**.

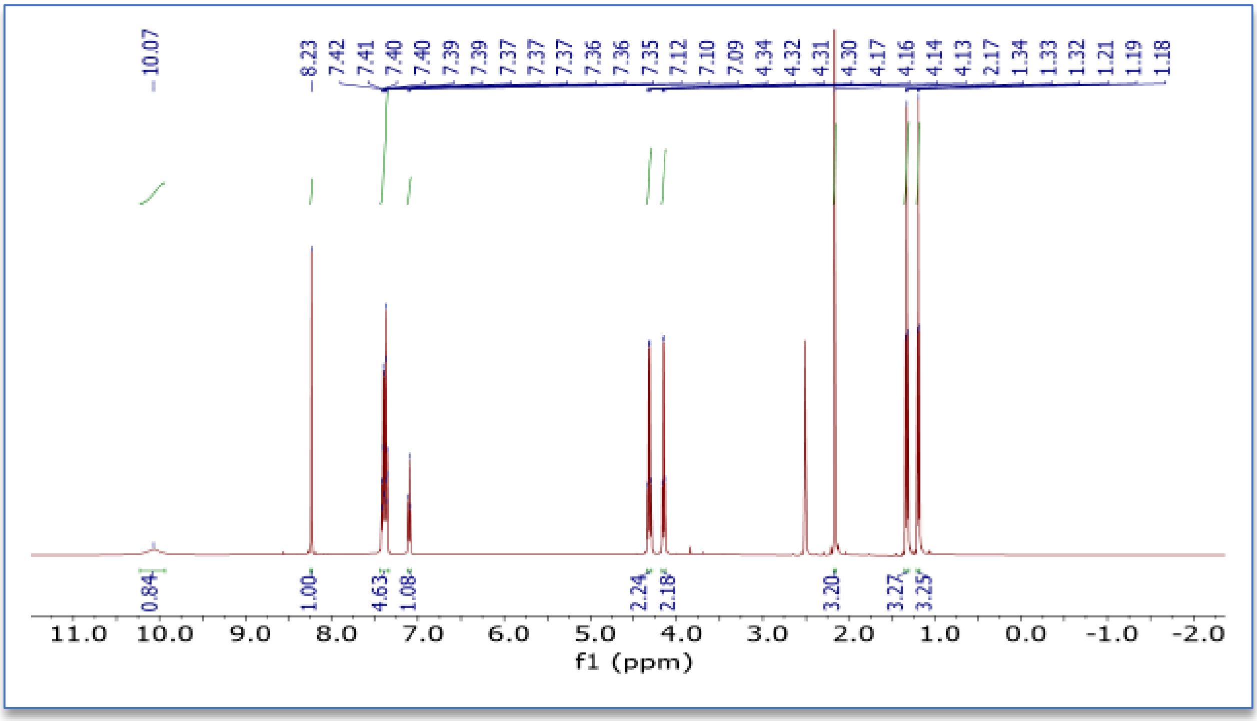

Fig. S9. ^{1}H NMR spectrum of **8b** at 400 MHz in DMSO-d_6.

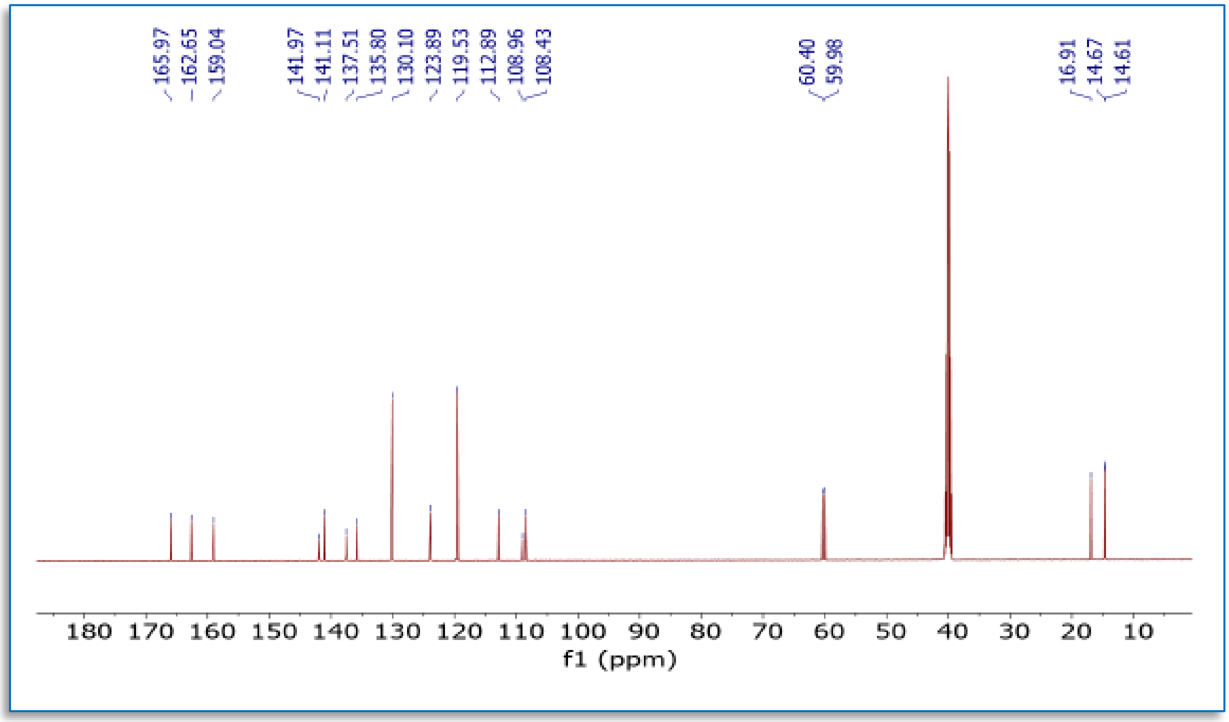

Fig. S10. ^{13}C NMR spectrum of **8b** at 100 MHz in DMSO-d_6.

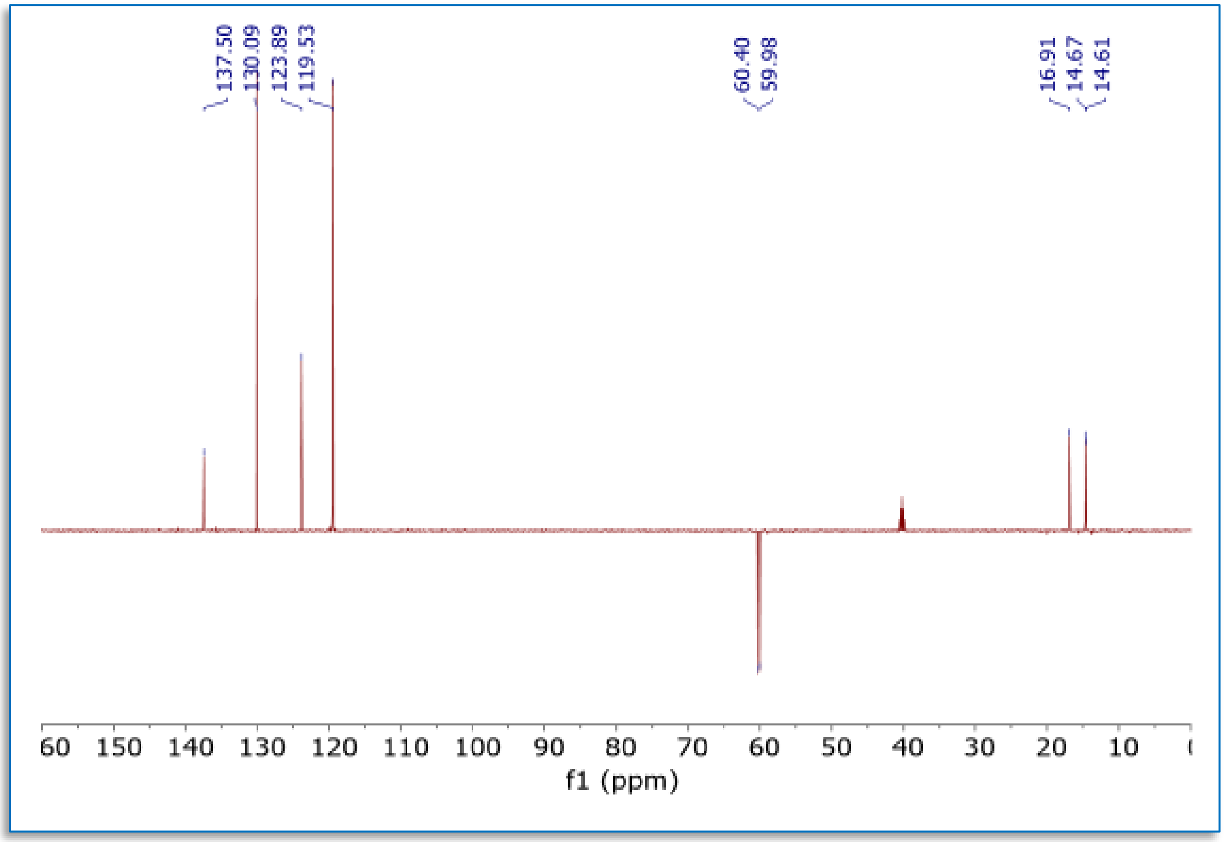

Fig. S11. ^{13}C NMR- DEPT-135 spectrum of **8b**.

Table S1. X-ray data of compound **8a**.

CCDC	2527582
Empirical formula	$C_{26}H_{25}N_3O_4S$
Formula weight, g/mol	475.55
Temperature/K	293
Crystal system	Monoclinic
Space group	P21/c (No. 14)
a/Å	11.3765(4)
b/Å	14.1839(5)
c/Å	15.3345(7)
α/°	90
β/°	107.680(4)
γ/°	90
Volume/Å^3	2357.55(17)
Z	4
ρ_{calc} g/cm^3	1.340
μ/mm^{-1}	0.176
F(000)	1000
Crystal size/mm^3	0.19 × 0.17 × 0.09
Crystal color, habit	Yellow, block
Radiation	MoKα (λ = 0.71073 Å)
2Θ range for data collection/°	6.27-54.89
Index ranges	$-14 \leq h \leq 14$, $-18 \leq k \leq 18$, $-19 \leq l \leq 18$
Reflections collected	19463
Independent reflections	5018
Goodness-of-fit on F^2 (S)	1.041
Final R indexes [I>=2σ (I)]	R_1 = 0.0853, wR_2 = 0.2244
wR2 [all data]	R_1 = 0.1242, wR_2 = 0.2942

4. CheckCIF data of compound 8a

checkCIF/PLATON report

You have not supplied any structure factors. As a result the full set of tests cannot be run.

THIS REPORT IS FOR GUIDANCE ONLY. IF USED AS PART OF A REVIEW PROCEDURE FOR PUBLICATION, IT SHOULD NOT REPLACE THE EXPERTISE OF AN EXPERIENCED CRYSTALLOGRAPHIC REFEREE.

No syntax errors found. CIF dictionary Interpreting this report

Datablock: rk-y36

```
Bond precision:  C-C = 0.0053 A          Wavelength=0.71073

Cell:            a=11.3765(4)    b=14.1839(5)        c=15.3345(7)
                 alpha=90        beta=107.680(4)     gamma=90
Temperature:     293 K

                Calculated                 Reported
Volume          2357.55(17)                2357.55(17)
Space group     P 21/c                     P 21/c
Hall group      -P 2ybc                    -P 2ybc
Moiety formula  C26 H25 N3 O4 S            C26 H25 N3 O4 S
Sum formula     C26 H25 N3 O4 S            C26 H25 N3 O4 S
Mr              475.55                     475.55
Dx,g cm-3       1.340                      1.340
Z               4                          4
Mu (mm-1)       0.176                      0.176
F000            1000.0                     1000.0
F000'           1000.92
h,k,lmax        14,18,19                   14,18,19
Nref            5389                       5018
Tmin,Tmax       0.967,0.984                0.677,1.000
Tmin'           0.967

Correction method= # Reported T Limits: Tmin=0.677
Tmax=1.000 AbsCorr = MULTI-SCAN

Data completeness= 0.931          Theta(max)= 27.444

R(reflections)= 0.0853( 3067)     wR2(reflections)= 0.2942( 5018)

S = 1.041                  Npar= 310
```

The following ALERTS were generated. Each ALERT has the format
test-name_ALERT_alert-type_alert-level.
Click on the hyperlinks for more details of the test.

Alert level C

```
RINTA01_ALERT_3_C   The value of Rint is greater than 0.12
            Rint given   0.159
PLAT020_ALERT_3_C The Value of Rint is Greater Than 0.12 .........      0.159 Report
PLAT084_ALERT_3_C High wR2 Value (i.e. > 0.25) ...................      0.29 Report
PLAT340_ALERT_3_C Low Bond Precision on  C-C Bonds ..............    0.00535 Ang.
```

Alert level G

```
PLAT007_ALERT_5_G Number of Unrefined Donor-H Atoms .............          1 Report
PLAT012_ALERT_1_G No       _shelx_res_checksum Found in CIF ......     Please Check
PLAT072_ALERT_2_G SHELXL First  Parameter in WGHT  Unusually Large       0.18 Report
PLAT199_ALERT_1_G Reported _cell_measurement_temperature ..... (K)        293 Check
PLAT200_ALERT_1_G Reported   _diffrn_ambient_temperature ..... (K)        293 Check
```

```
   0 ALERT level A = Most likely a serious problem - resolve or explain
   0 ALERT level B = A potentially serious problem, consider carefully
   4 ALERT level C = Check. Ensure it is not caused by an omission or oversight
   5 ALERT level G = General information/check it is not something unexpected

   3 ALERT type 1 CIF construction/syntax error, inconsistent or missing data
   1 ALERT type 2 Indicator that the structure model may be wrong or deficient
   4 ALERT type 3 Indicator that the structure quality may be low
   0 ALERT type 4 Improvement, methodology, query or suggestion
   1 ALERT type 5 Informative message, check
```

It is advisable to attempt to resolve as many as possible of the alerts in all categories. Often the minor alerts point to easily fixed oversights, errors and omissions in your CIF or refinement strategy, so attention to these fine details can be worthwhile. In order to resolve some of the more serious problems it may be necessary to carry out additional measurements or structure refinements. However, the purpose of your study may justify the reported deviations and the more serious of these should normally be commented upon in the discussion or experimental section of a paper or in the "special_details" fields of the CIF. checkCIF was carefully designed to identify outliers and unusual parameters, but every test has its limitations and alerts that are not important in a particular case may appear. Conversely, the absence of alerts does not guarantee there are no aspects of the results needing attention. It is up to the individual to critically assess their own results and, if necessary, seek expert advice.

Publication of your CIF in IUCr journals

A basic structural check has been run on your CIF. These basic checks will be run on all CIFs submitted for publication in IUCr journals (*Acta Crystallographica*, *Journal of Applied Crystallography*, *Journal of Synchrotron Radiation*); however, if you intend to submit to *Acta Crystallographica Section C* or *E* or *IUCrData*, you should make sure that full publication checks are run on the final version of your CIF prior to submission.

Publication of your CIF in other journals

Please refer to the *Notes for Authors* of the relevant journal for any special instructions relating to CIF submission.

PLATON version of 07/08/2019; check.def file version of 30/07/2019

Datablock rk-y36 - ellipsoid plot

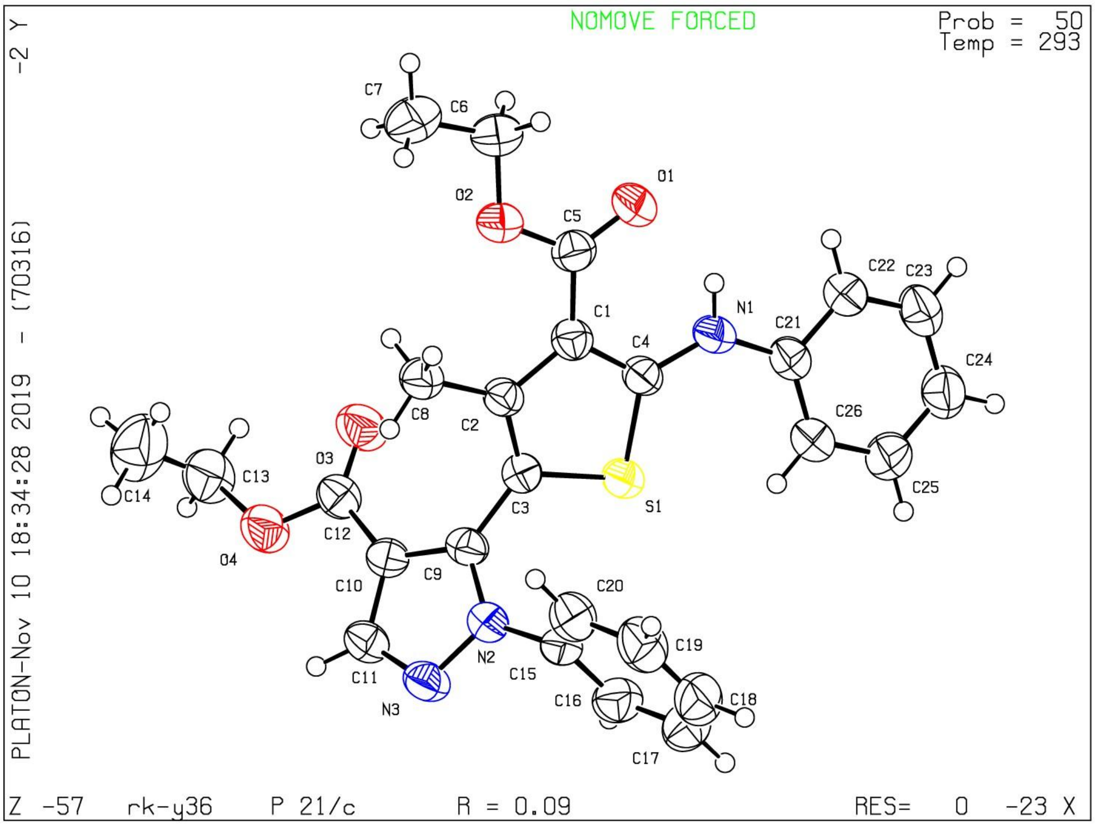

Supplement: Supplementary file 1 — Supplementary Material [file OPEN-15-e70226-s001.pdf]
